# Dental devices and antimicrobial resistance: challenges, innovations, and regulatory compliances

**DOI:** 10.3389/fmedt.2026.1750006

**Published:** 2026-03-20

**Authors:** Gargi Singh, Manav Singh, Anjanasree Murali, Sanyam Gandhi, Om V. Singh

**Affiliations:** 1 ITS Dental College and Research Centre, Ghaziabad, India; 2 Lucida Scientific and Regulatory Consulting, LLC, Laurel, MD, United States; 3 College of Computer, Mathematics, and Natural Sciences, Department of Biology, University of Maryland, College Park, MD, United States; 4 Diabetes, Medtronic MiniMed Inc., Northridge, CA, United States; 5 Regulatory Affairs Department, Takeda Pharma, Cambridge, MA, United States; 6 Advance Academic Programs, Krieger School of Arts and Sciences, Johns Hopkins University, Washington, DC, United States

**Keywords:** antimicrobial resistance (AMR), biofilm, dental devices, multidrug-resistant (MDR), oral resistome, regulatory compliance, risk management

## Abstract

Antimicrobial resistance (AMR) is an escalating threat to infection control in dentistry, where dense multispecies biofilms and frequent device-tissue contact can select for and disseminate antibiotic resistance genes (ARGs). The oral cavity functions as a reservoir and exchange network for ARGs (the oral resistome), enabling multidrug-resistant (MDR) infections and treatment failure, particularly on dental implants, restorative materials, and dental unit waterlines when reprocessing or maintenance is inadequate. Anti-biofilm surface engineering, including antimicrobial peptide-functionalized coatings, silver-based nanostructures, and nitric oxide-releasing platforms, offers non-antibiotic approaches to reduce adhesion and disrupt early biofilm development. However, translation requires addressing durability, long-term biocompatibility and toxicology (e.g., ion release), the potential for resistance or tolerance under sublethal exposure, and clinically meaningful validation in relevant multispecies models and human studies. Regulatory expectations (e.g., FDA quality system requirements under 21 CFR Part 820 and international standards ISO 13485, ISO 14971, and ISO 10993-1) frame risk management, antimicrobial performance claims, sterilization/reprocessing validation, and postmarket surveillance. Aligning antimicrobial stewardship with validated device design, sterilization, and monitoring strategies is essential to mitigate AMR proliferation and improve long-term clinical outcomes. This article is a comprehensive narrative review focusing on AMR in dental devices, materials innovation, and regulatory frameworks.

## 1 Introduction

Antimicrobial resistance (AMR) is the ability of microorganisms to survive and proliferate despite antibiotic exposure. It is increasingly relevant to dentistry because the oral cavity supports dense, multispecies biofilms where resistance can persist, transfer, and re-emerge after therapy. Misuse and overuse of antibiotics in medicine and dentistry, together with environmental antimicrobial exposure, accelerate selection for resistant strains and mobilization of antibiotic resistance genes (ARGs) (1, 2).

Rising resistance among pathogens that also occur in oral and healthcare environments (e.g., *Staphylococcus aureus, Pseudomonas aeruginosa, Enterococcus faecalis*) increases the likelihood of persistent or recurrent infections (3–5), particularly when biofilms reduce antimicrobial penetration and enhance horizontal gene transfer. Device-associated infections are especially challenging because systemic antibiotics often have limited efficacy against established biofilms and may further select for resistance.

In dentistry, infection prevention depends on validated reprocessing and waterline maintenance, clear labeling for cleaning/sterilization, and risk-based device design. From a regulatory perspective, antimicrobial risk is addressed through design controls, sterilization and reprocessing validation, biocompatibility evaluation, and postmarket surveillance within quality and risk management frameworks. Globally, the international standards (ISO 13485, ISO 14971, ISO 10993, ISO 11135, ISO 17665) ensures safety, performance, and lifecycle risk management of the dental devices. Whereas the role of the European Medicines Agency (EMA) and the EU Medical Device Regulation [MDR, Regulation (EU) 2017/745] governs high-risk dental and implantable devices, particularly in relation to post-market surveillance, clinical evidence, and antimicrobial stewardship. This review links oral resistome evidence with device-associated infection risks, summarizes emerging anti-biofilm materials and smart surfaces, and maps these innovations to regulatory expectations for safety, effectiveness, and lifecycle control.

## 2 Human oral microbiome and multidrug resistance

**Figure 1:**
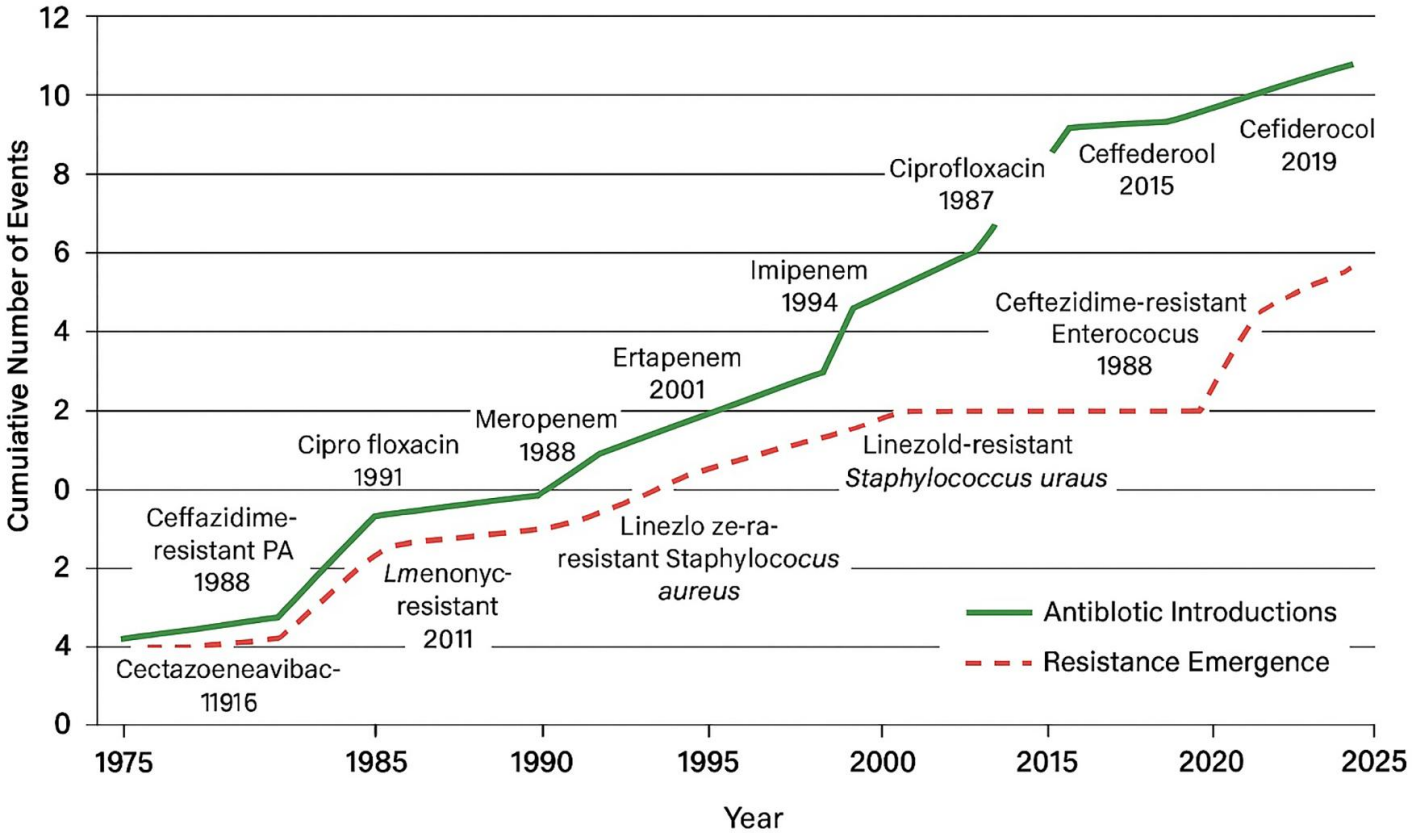
Antibiotic Introductions and Resistance Emergence (1975–2025). (The figure summarizes major antibiotic introductions (green line) and the corresponding first reports of bacterial resistance (red dashed line). Data derived from Davies & Davies (6), Fair & Tor (7), CDC (8), Bush & Bradford (9), and WHO (10).

The human oral microbiome is a complex, site-specific community that includes bacteria, fungi, viruses, and archaea. Its AMR relevance lies less in taxonomic breadth than in the biofilm lifestyle: oral microorganisms form structured biofilms on teeth, soft tissues, and device surfaces, creating diffusion barriers and microenvironments that promote persistence and antimicrobial tolerance. Historically, the emergence of new antibiotics has been swiftly followed by the development of corresponding resistance mechanisms, often within a few years of clinical introduction (Figure 1).

Metagenomic and culture-based studies show that the oral cavity can act as a reservoir for ARGs, including determinants relevant to beta-lactams, macrolide-lincosamide-streptogramins, and tetracyclines, and that resistome composition varies across niches and clinical states (11–14). Clinically, ARG carriage may occur without overt disease yet can contribute to peri-implantitis, endodontic infections, and healthcare-associated transmission when biofilms form on devices or when reprocessing is inadequate.

These findings underscore why biofilm-aware infection control and antimicrobial stewardship must be paired with device strategies that reduce bacterial adhesion and improve cleanability and reprocessability, especially for implantable and reusable devices.

## 3 Bioinformatics of antibiotic resistance oral microbiome

When exposed to selective environmental pressures, microorganisms can activate or acquire antibiotic resistance genes (ARGs) to enhance their survival and ensure genetic persistence. Consequently, the oral cavity serves as an important reservoir for ARGs, increasing the likelihood of antibiotic-resistant infections (15, 16). The Human Oral Microbiome Database (HOMD) was the first systematic effort to catalogue a human-associated microbiome, providing analytical tools to help researchers investigate the relationships between microbial communities and health outcomes. This database compiles detailed information on approximately 700 bacterial species inhabiting the oral cavity, following a curated taxonomy based on 16S rRNA gene sequences. Over the past two decades, more than 600 16S rRNA gene libraries have been generated, yielding over 35,000 clone sequences (17). The expanded version, known as the extended Human Oral Microbiome Database (eHOMD), further includes microbial species found in both the oral and nasal cavities (18).

Several studies have expanded understanding of the oral resistome and its relation to health and disease. Almeida et al. (19) examined the diversity of the oral microbiome and prevalence of ARGs in individuals with healthy and diseased periodontal tissues using 16S rRNA gene analysis. The study revealed that healthy individuals exhibited greater microbial diversity than those with disease, although the overall proportion of ARGs remained similar between the two groups. The predominant resistance genes identified were *erm, blaTEM, mecA, and pbp2b*, suggesting horizontal gene transfer among oral bacteria. Sukumar et al. (20) investigated the development of the pediatric oral resistome and its association with dental caries in 221 twin children sampled across the first decade of life. Analysis of 530 oral metagenomes identified 309 ARGs, which clustered by age and showed genetic influence from infancy onward. The findings suggested that ARG mobilization increases with age, supported by the observation that the mobile genetic element Tn916 transposase co-localized with a larger number of species and ARGs in older children.

Anderson et al. (21) explored the resistome and phenotypic antibiotic resistance profiles of oral biofilm microbiota from 179 individuals with healthy, caries-active, and periodontally diseased conditions. The authors identified 64 ARGs conferring resistance to 36 antibiotics, particularly those in the tetracycline, macrolide-lincosamide-streptogramin, and beta-lactam classes. A higher prevalence of ARGs was found in samples from healthy and caries-active participants compared with those with periodontal disease, and distinct resistotypes were observed based on microbial composition.

At the metagenomic scale, Zhu et al. (22) assembled 56,213 high- and medium-quality metagenome-assembled genomes (MAGs), which, along with 190,000 public genomes, were clustered into 3,589 oral species-level clades. This comprehensive genomic dataset provides an important reference resource for culture-based and functional studies, as well as diagnostic and therapeutic developments related to the oral microbiome.

In recent years, additional curated platforms have been established. The Comprehensive Antibiotic Resistance Database (CARD) provides an authoritative source that integrates gene sequences, resistance mechanisms, and ontology-based annotations for detailed resistome characterization (23). ResFinder and PointFinder focus on identifying acquired resistance genes and chromosomal mutations directly from genomic data (24). For large-scale metagenomic studies, the MEGARes database offers a non-redundant, hierarchically organized collection of resistance genes optimized for quantitative statistical analyses (25). The National Database of Antibiotic-Resistant Organisms (NDARO), managed by the U.S. National Center for Biotechnology Information (NCBI), provides centralized genomic surveillance data and standardized resistance profiles for clinically relevant bacteria (26). Table 1 summarizes Major ARG Databases Applicable to Oral Microbiome and Dental Device Research that may be useful to practitioners and scientists with research interests in antimicrobial resistance of oral and Dental research.

**Table 1:** Major antibiotic resistance gene (ARG) databases applicable to oral microbiome and dental device research.

Database/ Version	Relevance to oral/dental	Genes/Genomes	Website	Features	Challenges	References
eHOMD/HOMD (Expanded Human Oral Microbiome Database)/Version 4 (2025)	Core reference for oral bacterial taxonomy and genome annotation; used for mapping ARGs to oral taxa and dental niches	∼834 taxa (oral/nasal); hundreds of whole genomes	https://www.homd.org	Curated taxonomy, 16S and whole-genome linkages, BLAST support, genome browser	Does not include intrinsic ARG annotations; must be cross-referenced with external ARG databases	(18, 27)
CARD (Comprehensive Antibiotic Resistance Database)/Continuous (2023 update)	Frequently used to annotate ARGs from oral and dental-device samples	>6,000 reference sequences, including SNP-based models	https://card.mcmaster.ca	Mechanism-based curation, ontology-linked (ARO), phenotype associations	May underrepresent oral niche–specific ARGs or mobile genes	(23)
ResFinder/PointFinder/ResFinder v4.x	Commonly applied for dental and periodontal bacterial isolates	Varies by species; extensive ARG and mutation data	https://cge.food.dtu.dk/services/ResFinder	Links genotype to phenotype; identifies acquired genes and mutations	Limited for novel ARGs; less suitable for complex biofilm resistomes	(24)
MEGARes/AMR++/Version 3.0 (2022)	Suitable for metagenomic oral biofilm and plaque resistome profiling	∼8,700 curated resistance genes	https://megares.meglab.org	Non-redundant hierarchical structure; compatible with AMR++ pipeline	Requires integration with taxonomy databases for oral-specific analyses	(28)
FARME (Functional Antibiotic Resistance Metagenomic Element DB)/Ongoing (multiple metagenomic projects)	Captures functional ARGs from uncultured or novel oral bacteria	Functionally validated ARGs and mobile elements	Via publications/project sites	Identifies ARGs from functional metagenomics of oral biofilms	Requires manual verification; limited clinical correlation	(29)
INTEGRALL/Release 1.2 (2021)	Catalogues integrons and gene cassettes linked to oral biofilm-mediated ARG transfer	∼12,000 integron entries, ∼8,500 gene cassettes	https://integrall.bio.ua.pt	Focused on integrons and horizontal gene transfer elements	Does not include full ARG catalog; mainly integron-related	(30)
NDARO (National Database of Antibiotic Resistant Organisms)/Continuous (2024)	Supports genomic surveillance of oral pathogens and hospital-acquired dental infections	Thousands of genomes; linked phenotype data	https://www.ncbi.nlm.nih.gov/pathogens/antimicrobial-resistance	Centralized U.S. AMR surveillance; integrates genotype and phenotype	Primarily clinical isolates; limited environmental/dental coverage	(26)
ARGminer/2019 release (actively maintained)	Crowdsourced ARG integration, including oral/environmental sources	>20,000 entries aggregated from multiple ARG databases	https://bench.cs.vt.edu/argminer	Harmonizes ARG nomenclature; cross-links multiple ARG sources (CARD, ARDB, NDARO)	Requires manual verification; variable annotation consistency	(31)
Local/Study-Specific Oral Resistome Catalogs/Based on publication year	Custom ARG sets derived from oral plaque, dental devices, and peri-implant infections	Varies (dozens–hundreds of ARGs per study)	Available in supplementary materials	Reflect real-world ARG diversity in dental niches	Not standardized or widely integrated; limited comparability	(20–23)

Together, these bioinformatics resources—supported by advances in computational genomics and open data sharing—enable rapid access to curated, up-to-date genetic and phenotypic information. This accessibility enhances antimicrobial resistance research, supports global surveillance efforts, and facilitates the development of novel antimicrobial strategies across medical, dental, and environmental contexts.

Clinical and regulatory relevance of oral resistome data: Resistome profiling can support antimicrobial stewardship by informing empiric therapy and by identifying high-risk ARG signatures in settings with frequent device use (e.g., implant dentistry and endodontics). For device developers and regulators, oral resistome datasets help define clinically plausible challenge organisms and resistance phenotypes for antimicrobial performance testing, guide risk management plans (ISO 14971), and motivate postmarket monitoring strategies when antimicrobial claims are made or when real-world use could promote biofilm persistence. Importantly, presence of ARGs does not automatically predict clinical failure; therefore, resistome evidence should be interpreted alongside clinical outcomes, exposure history, and biofilm context when setting performance endpoints.

## 4 Infection transmission in dental care clinics

Dental procedures routinely generate droplets and aerosols and involve contact with saliva and blood, enabling transmission through inhalation, mucosal exposure, direct contact, and contaminated instruments or environmental surfaces (32–36). Bioaerosols can settle on operatory surfaces and equipment, and viable microorganisms may persist for extended periods, making timely cleaning and disinfection essential (36, 37).

Risk reduction is most effective when controls are layered: engineering controls (high-volume evacuation, extraoral suction, ventilation), standardized surface decontamination, validated instrument reprocessing, and appropriate personal protective equipment. Because transmission risks intersect with device design and reprocessing, clear instructions for use (IFU), sterilization/reprocessing validation, and routine monitoring (e.g., dental unit waterline testing and maintenance) are critical to prevent amplification of MDR organisms within clinics and to support compliance with quality system and infection control expectations.

## 5 Infections led by dental devices

**Figure 2:**
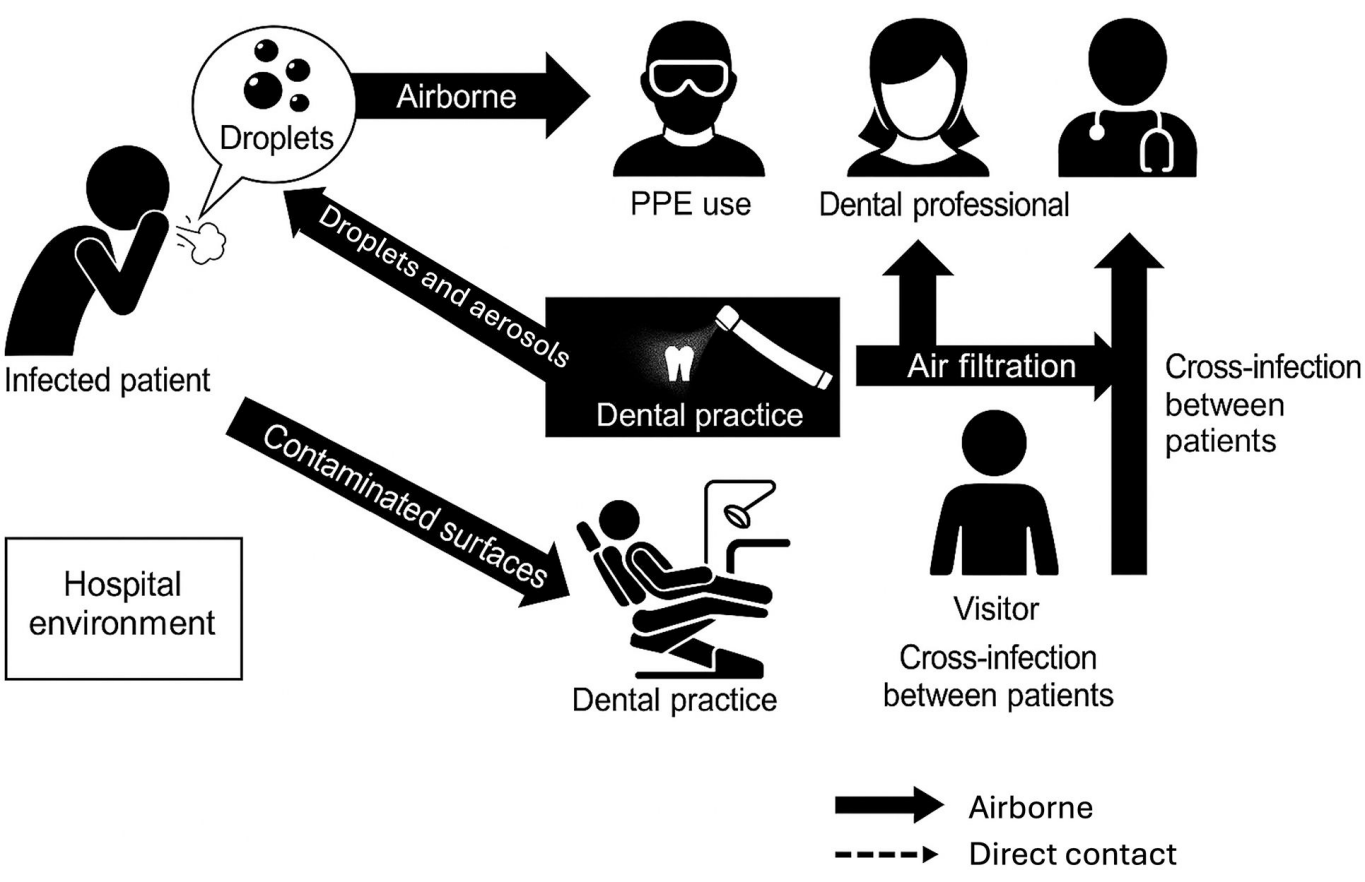
Transmission routes of pathogens in dental care clinics and hospital environments (Schematic representation of the primary routes of pathogen transmission in dental care environments, including airborne, droplet, contact, and fomite-mediated pathways. Pathogens may spread via inhalation of aerosolized microorganisms, direct exposure to blood and saliva, contact with contaminated instruments or environmental surfaces, and splatter from high-speed dental equipment. The diagram highlights the role of dental professionals, patients, and environmental factors in cross-infection risk. Adapted from Peng et al. (38) and modified with other findings by Kampf et al. (39), CDC (33), and Yang et al. (40).

Healthcare-associated infections (HAIs) caused by multidrug-resistant (MDR) pathogens are increasing worldwide, while current antimicrobial strategies often show limited efficacy against these resilient organisms. In dental environments, both patients and personnel are routinely exposed to a diverse array of microorganisms found in blood, saliva, and aerosols produced during clinical care. These include *Mycobacterium tuberculosis, hepatitis B virus, Staphylococcus spp., Streptococcus spp., cytomegalovirus*, herpes simplex virus types I and II, human T-lymphotropic virus type III/lymphadenopathy-associated virus (HTLV-III/LAV), and several respiratory pathogens. Transmission can occur through direct contact with contaminated blood or saliva, inhalation of infectious droplets and aerosols, or indirect contact via contaminated instruments and surfaces. Both patients and dental healthcare workers (DHCWs) can act as sources or recipients of infection (Figure 2).

Dental and medical implants have become indispensable for restoring anatomical and functional integrity in the oral cavity. These biomaterials—commonly made of metals, ceramics, or composite polymers—are, however, prone to microbial colonization. Following placement, microorganisms form structured biofilms embedded within an extracellular polymeric substance (EPS) matrix. Biofilms on implant surfaces typically contain both Gram-positive and Gram-negative pathogens such as *Enterococcus faecalis*, *Escherichia coli, Klebsiella pneumoniae, Staphylococcus aureus, Staphylococcus epidermidis, Streptococcus viridans,* and *Pseudomonas aeruginosa* (41, 42). These communities are notoriously resistant to antimicrobial therapy and immune clearance, often resulting in chronic infections, inflammation, and eventual implant failure (43).

Dental implants are now a standard of care for tooth replacement (44). Under healthy conditions, the peri-implant microbiota resembles that of natural teeth, dominated by Gram-positive cocci and rods (44). However, peri-implant diseases—specifically peri-implant mucositis and peri-implantitis—remain significant clinical concerns. Peri-implantitis prevalence is estimated to affect approximately 12%–20% of implants and up to 24% of patients globally (45, 46). The condition is characterized by microbial biofilm accumulation, tissue inflammation, and progressive bone loss around implants (47, 48). Early-stage peri-implant mucositis manifests as erythema, edema, and bleeding of peri-implant soft tissue, which, if untreated, may progress to peri-implantitis, leading to irreversible bone resorption and implant instability.

Odontogenic sinusitis (ODS) also represents an infection type linked to dental procedures and implant interventions. ODS usually originates in the maxillary sinus through bacterial migration from periapical or endodontic sources, or iatrogenic compromise of the Schneiderian membrane (49). Maxillary sinus grafting (MSG), often performed to increase bone volume for osseointegration, carries a risk of infection when microbial contamination occurs in either the graft or the overlying sinus. In severe cases, infection may extend across the sinus mucosa, resulting in secondary sinusitis if early intervention is not achieved.

Dental operative units and their associated waterlines are another potential source of microbial exposure. These systems deliver air, water, and power to dental instruments such as handpieces and ultrasonic scalers. Dental unit waterlines (DUWLs), typically composed of polymers like polyurethane or polyvinyl chloride, can support biofilm formation if maintenance is inadequate. While municipal water generally meets drinking standards, it may harbor opportunistic pathogens such as *Pseudomonas aeruginosa*, *Legionella pneumophila*, and *Mycobacterium* spp., which pose infection risks during dental procedures (50). Microorganisms can adhere to waterline surfaces, form biofilms, and subsequently release cells into the water stream, contaminating the cooling and irrigation water used intraorally (51).

To mitigate these risks, the American Dental Association (ADA) and the Centers for Disease Control and Prevention (CDC) recommend maintaining bacterial counts in dental unit water at ≤500 colony-forming units (CFU) per milliliter of heterotrophic bacteria. Recent studies emphasize the need for regular microbial testing and disinfection protocols, including shock treatments and continuous antimicrobial dosing to reduce biofilm reformation (52, 53). Sampling should include points at the water source, the handpiece outlet, and midline tubing sections. Consistent monitoring, use of filtered or distilled water, and adoption of antimicrobial tubing materials can substantially reduce the risk of DUWL-related infections and enhance patient safety.

## 6 Strategies to control device-associated infection

Preventing implant-related infections is far more effective than managing them after onset. Modifying implant surfaces offers an effective strategy to minimize bacterial adhesion, eradicate adherent microorganisms, and prevent biofilm formation, thereby improving long-term clinical outcomes (43). Antibacterial approaches can be categorized as passive or active. Passive strategies involve altering surface topography, wettability, and charge to hinder bacterial adhesion and biofilm initiation. Whereas active strategies rely on incorporating or releasing antimicrobial agents such as peptides, metals, polymers, or antibiotics to directly kill microbes (52, 53). Emerging stimuli-responsive (“smart”) surfaces activate their antimicrobial function only when triggered by local or external cues such as pH changes, enzymes, temperature, or light exposure (54). These coatings allow on-demand antimicrobial activity, preventing premature depletion while maintaining long-term effectiveness. Improved osseointegration further reduces infection risk by physically limiting bacterial colonization (46).

Translational considerations and limitations: While antimicrobial coatings and smart, stimuli-responsive surfaces show promise, their clinical translation is constrained by (i) durability under mechanical wear and repeated sterilization/reprocessing; (ii) long-term biocompatibility and toxicology, including cumulative ion or drug release (ISO 10993); (iii) the risk of resistance or tolerance under sublethal, sustained exposure; and (iv) inconsistent testing models. Many studies rely on short-term, single-species or static biofilm assays that do not capture multispecies oral biofilms, salivary pellicle effects, pH fluctuations, or shear forces. Standardized, regulator-acceptable test methods and clinically relevant endpoints (e.g., reduction in biofilm burden linked to peri-implant outcomes), supported by appropriate bench, animal, and human evidence, are critical for substantiating antimicrobial performance claims and selecting feasible regulatory pathways [510(k), *de novo*, or PMA, depending on risk and claims].

### 6.1 Antimicrobial coatings

Self-defensive antimicrobial coatings remain inactive under normal physiological conditions and are triggered only in the presence of bacterial growth. Pathogenic bacteria release organic acids or virulence-related enzymes that alter local pH, initiating antimicrobial release or coating degradation—a process sometimes called self-polishing (55). Such coatings can also be externally activated by heat, light, or electromagnetic fields, producing a localized, controlled antibacterial response that preserves coating longevity (56).

### 6.2 Antimicrobial peptides (AMPs)

AMPs are short cationic peptides (12–50 amino acids) exhibiting broad-spectrum antimicrobial activity against both Gram-positive and Gram-negative bacteria (57, 58). Their primary mechanism involves electrostatic disruption of bacterial membranes, resulting in lysis (59). Recent research confirms that AMP coatings on titanium or zirconia implants inhibit *Staphylococcus aureus* and *Porphyromonas gingivalis* biofilms while supporting osteoblast adhesion (60, 61). AMPs such as GL13K and Tet213 have achieved > 99% bacterial inhibition, showing promise for peri-implant infection prevention (62). A summary of recent AMP-based coatings, bacterial targets, and outcomes is presented in Table 2.

**Table 2:** Antimicrobial peptide (AMP) functionalization of Titanium dental implant surfaces: *in vitro* antibacterial activity and release characteristics.

AMP	Substrate	Follow-up	Bacterial culture	AA/BC/BS	Results	Release	References
Cys–GL13K (chemoselective)	Titanium (silane chemistry)	Mechanical challenge + biofilm tests	Oral pathogens	AA, BC	Stable coating post-challenge; biofilm suppression	Immobilized	(61, 63)
hBD-3	Titanium (nano/porous)	7 d	*S. aureus, E. coli*	BC, BS	Significant killing (*p* < 0.005)	Slow & sustained	(64)
GL13K (silanized)	Titanium (silanization)	24–72 h	Macrophage–material interactions; peri-implant pathogens	—	Anti-inflammatory polarization with antibacterial intent	Non-eluting	(63)
hBD-3 (loaded)	Titanium	24–72 h	Planktonic & sessile bacteria	BC	Robust antimicrobial effect on Ti	Controlled release	(65)
LL-37–hyaluronic acid conjugate	Titanium	24–72 h	*S. aureus*	BC	Hybrid organic coating shows antibacterial activity	Gradual (HA matrix)	(66)
ε-Polylysine (± chitosan)	Anodized titanium	24–72 h	Oral/enteric models	AA, BC	Improved antibacterial + biocompatibility vs. anodized Ti	Gradual from polymer matrix	(67)

AA, anti-adherent; BC, bactericidal; BS, bacteriostatic.

### 6.3 Synthetic and non-antibiotic molecules

In addition to AMPs, various polymers, antibiotics, and non-antibiotic organic antimicrobials (NOAs) have been immobilized on implant surfaces to reduce bacterial colonization. Poly(ethylene glycol) (PEG) coatings form hydrophilic layers that minimize microbial adhesion (68). Chlorhexidine (CHX)–based nanoparticle coatings (CHX-HMP) enable sustained antibacterial release against *Streptococcus gordonii* and complex oral biofilms (69, 70). Polymeric nanofibers loaded with tetracycline or doxycycline also provide prolonged activity against peri-implant pathogens such as *Fusobacterium nucleatum* and *P. gingivalis* (71). However, excessive antibiotic release may induce cytotoxicity and resistance; thus, hybrid coatings combining chitosan with inorganic agents like zinc oxide are gaining traction for their dual antibacterial and biocompatible behavior (72). Representative synthetic and non-antibiotic antimicrobial coatings are summarized in Table 3.

**Table 3:** Non-Peptide antimicrobial coatings and molecule-based surface modifications on Titanium dental implants: *in vitro* antibacterial efficacy and release characteristics.

Molecule/Strategy	Substrate	Follow-up	Bacterial Culture	AA/BC/BS	Key Results	Release Behavior	References
CHX–HMP	Titanium	24–72 h	*Streptococci*	BS/AA	CHX-HMP coatings inhibit biofilm formation and show cytocompatibility.	Early burst; minimal after 14 days.	(70)
TESPSA	Titanium (varied roughness)	12–24 h	Multispecies oral biofilm	AA	Reduced biofilm adhesion with preserved cell viability.	Covalently immobilized; non-releasing.	(73)
Chitosan + ZnO	Titanium	24–72 h	*S. aureus, E. coli*	BC	Hybrid coating improves antibacterial and mechanical properties.	Contact-active; ZnO driven.	(74)
Chitosan/ZnO composite	3D-printed Titanium	24–72 h	Peri-implant pathogens	BC/AA	Improved antimicrobial, adhesion, and mechanical properties.	Primarily contact-active; minimal elution.	(56)
Minocycline on GO	Titanium (graphene oxide interlayer)	24–72 h	*S. aureus*	BC	Synergistic contact-killing + sustained antibiotic release.	Slow, sustained release from GO layer.	(75)
Gentamicin (collagen/HA LbL)	Titanium	10 days	*S. aureus, E. coli*	BC	Effective bacterial reduction with cytocompatibility.	Sustained release (∼240 h).	(76)

AA, anti-adherent; BC, bactericidal; BS, bacteriostatic.

### 6.4 Silver nanoparticles (AgNPs)

Metal nanoparticles—especially silver, copper, and zinc—have long been recognized for their antimicrobial potency. Silver nanoparticles (AgNPs) remain the most studied for biomedical use because of their strong bactericidal, antifungal, and anti-biofilm activity (77, 78). Their mechanisms include membrane disruption, enzyme inhibition, oxidative stress generation, and interference with bacterial replication (79). AgNP-modified titanium and silica coatings have demonstrated bacterial survival reductions of 40%–60% compared with unmodified controls (80). Because cytotoxicity depends on nanoparticle morphology and dosage, hybrid coatings that combine AgNPs with polymers or ceramics are being developed to balance antimicrobial efficacy and tissue compatibility.

## 7 Dental devices and regulatory compliances

### 7.1 Spectrum of dental devices

Dental devices encompass a wide range of tools, instruments, materials, and technologies utilized in the prevention, diagnosis, and management of oral and maxillofacial conditions. These devices form the backbone of contemporary dental practice, supporting precision, enhancing clinical outcomes, and improving patient safety (81). Their use spans clinical, academic, and industrial contexts, and they are generally classified according to their intended purpose into diagnostic, preventive, restorative, surgical, implantable, and orthodontic categories.

Diagnostic devices—including digital radiography, intraoral cameras, and cone-beam computed tomography (CBCT)—are vital for early disease detection, risk assessment, and treatment planning (82, 83). Preventive devices, such as fluoride trays, sealant applicators, and mouthguards, play an essential role in mitigating caries development and protecting against oral trauma (84). Restorative and prosthodontic devices, including composite resins, Computer-aided design and Computer-aided manufacturing (CAD/CAM) systems, and zirconia-based restorations, are used to restore dental form and function, supported by advances in adhesive technology and digital design (85, 86).

Surgical devices, such as ultrasonic scalers, laser systems, and bone drills, are crucial in periodontal, implant, and oral surgical procedures, with laser-assisted systems offering superior precision and minimal tissue trauma (87). Implantable dental devices, especially titanium and zirconia implants, remain the standard for oral rehabilitation due to their excellent biocompatibility, corrosion resistance, and osseointegration potential (88, 89). Orthodontic systems, including metal and ceramic brackets as well as clear aligners such as Invisalign®, are increasingly guided by digital workflows, allowing for high precision in tooth movement and enhanced patient comfort (90).

Recent technological progress continues to redefine the dental device landscape. Digital dentistry—through intraoral scanning, 3D printing, and CAD/CAM milling—has improved efficiency, customization, and the accuracy of prosthetic and surgical solutions (86, 90). Artificial intelligence (AI) and machine learning algorithms are being integrated into diagnostic imaging, caries detection, and predictive analytics to enhance clinical decision-making and automate treatment workflows (82, 83). Meanwhile, smart dental devices—such as Bluetooth-enabled toothbrushes, salivary biosensors, and intraoral wearables—are part of a rapidly growing field aimed at real-time monitoring of oral health (91–93).

Material innovation remains central to the development of dental devices. Metals such as titanium and stainless steel, ceramics like zirconia and porcelain, and polymers including polymethyl methacrylate (PMMA) and composite resins are widely employed due to their mechanical strength and biocompatibility. Compliance with ISO 10993 ensures the biological safety of materials, while ISO 13485 and Good Manufacturing Practices (GMPs) regulate quality management and production consistency (94, 95).

The integration of additive manufacturing has further transformed the dental industry by enabling rapid prototyping and the fabrication of patient-specific devices such as surgical guides, orthodontic aligners, and crowns (96, 97). These technologies not only reduce production time and cost but also enhance precision and sustainability. As digitalization and biomaterial science continue to converge, the global dental device industry is moving toward greater personalization, regulatory harmonization, and sustainability—ultimately advancing patient-centered care and clinical efficiency.

### 7.2 Regulatory classification and compliances of dental devices

Dental devices are regulated globally under risk-based frameworks to ensure safety, performance, and postmarket vigilance. In the United States, the Food and Drug Administration (FDA) classifies dental devices according to risk under Title 21 of the Code of Federal Regulations (CFR) Part 872, which governs dental instruments, materials, and accessories. Devices are assigned to Class I (low risk), Class II (moderate risk), or Class III (high risk), with most dental devices falling within Class I or II (98). Class III devices, such as endosseous dental implants (21 CFR 872.3640) and bone grafting materials (21 CFR 872.3930), require Premarket Approval (PMA) due to their higher risk profile and their direct interface with human tissues. Table 4 provides comprehensive list of all the medical dental devices that are being regulated by the US FDA. Compliance with 21 CFR Part 820 (Quality System Regulation), ISO 13485 (Quality Management Systems), and ISO 14971 (Risk Management) remains mandatory for all manufacturers (95, 99).

**Table 4:** FDA classification of dental devices under 21 CFR part 872: diagnostic, prosthetic, surgical, therapeutic, and miscellaneous categories^b^.

FDA regulation		Device types	Class	Product code
21CFR 872				
Subpart B – Diagnostic Devices	*Sec.*			
	872.1500	Gingival fluid measurer	I	JEO
	872.1720	Pulp tester	II^a^	EAT
	872.1730	Electrode gel for pulp testers	I	EAS
	872.1740	Caries detection device	II	LFC, NYH
	872.1745	Laser fluorescence caries detection device		NBL, NTK
	872.1800	Extraoral source x-ray system		EHD, MUH
	872.1810	Intraoral source x-ray system		EAP
	872.1820	Dental x-ray exposure alignment device	I	EHA
	872.1830	Cephalometer	II	EAG
	872.1840	Dental x-ray position indicating device	I	EHB
	872.1850	Lead-lined position indicator		EAH
	872.1870	Sulfide detection device	II	MVH
	872.1905	Dental x-ray film holder	I	EGZ
	872.2050	Dental sonography device	II	NFP, NFQ
	872.2060	Jaw tracking device	I & II^a^	NFR, NFS
Subpart C	Reserved			
Subpart D - Prosthetic Devices	872.3060	Noble metal alloy	II^a^	EIT, EJS, EJT
	872.3070	Dental amalgam, mercury, and amalgam alloy		EJJ, ELY, OIV
	872.3080	Mercury and alloy dispenser	I	EHE
	872.3100	Dental amalgamator		EFD
	872.3110	Dental amalgam capsule		DZS
	872.3130	Preformed anchor		EJX
	872.3140	Resin applicator		KXR
	872.3150	Articulator		EJP, KZO
	872.3165	Precision attachment		EGG, EHO
	872.3200	Resin tooth bonding agent	II	KLE
	872.3220	Facebow	I	KCR
	872.3240	Dental bur		EJL, NME
	872.3250	Calcium hydroxide cavity liner	II	EJK
	872.3260	Cavity varnish	II^a^	LBH, PHR, PME
	872.3275	Dental cement	I & II	EMA, MZW, NEA, EMB
	872.3285	Preformed clasp	I	EHP. EJW
	872.3300	Hydrophilic resin coating for dentures	II	EBE
	872.3310	Coating material for resin fillings		EBD
	872.3330	Preformed crown	I	ELZ
	872.3350	Gold or stainless steel cusp		ELO
	872.3360	Preformed cusp		EHQ
	872.3400	Karaya and sodium borate with or without acacia denture adhesive	I & III	LOR, MMU, KOM
	872.3410	Ethylene oxide homopolymer and/or carboxymethylcellulose sodium denture adhesive	I	KOL, KOQ, KXW
	872.3420	Carboxymethylcellulose sodium and cationic polyacrylamide polymer denture adhesive	III	KOS
	872.3450	Ethylene oxide homopolymer and/or karaya denture adhesive	I	KOP, KXX
	872.3480	Polyacrylamide polymer (modified cationic) denture adhesive	III	KON
	872.3490	Carboxymethylcellulose sodium and/or polyvinylmethylether maleic acid calcium-sodium double salt denture adhesive	I	KOO, KOT
	872.3500	Polyvinylmethylether maleic anhydride (PVM-MA), acid copolymer, and carboxymethylcellulose sodium (NACMC) denture adhesive	III	KXY
	872.3520	OTC (Over the Counter) denture cleanser	I	EFT, NUX
	872.3530	Mechanical denture cleaner		JER
	872.3540	OTC denture cushion or pad	I & II^a^	HER, EHS, NKJ
	872.3560	OTC denture reliner	II^a^	EBP
	872.3570	OTC denture repair kit	II	EBO
	872.3580	Preformed gold denture tooth	I	ELN
	872.3590	Preformed plastic denture tooth	II^a^	ELM, PZY
	872.3600	Partially fabricated denture kit		EKO
	872.3630	Endosseous dental implant abutment		NHA, PNP
	872.3640	Endosseous dental implant		DZE, NRQ, OAT
	872.3645	Subperiosteal implant material	II	ELE
	872.3660	Impression material	II^a^	ELW
	872.3661	Optical Impression Systems for CAD/CAM		KZN, NOF, QJK
	72.3670	Resin impression tray material	I	EBH
	872.3680	Polytetrafluoroethylene (PTFE) vitreous carbon materials	II	NFE
	872.3690	Tooth shade resin material		EBF, OFW
	872.3710	Base metal alloy	II^a^	EJH
	872.3730	Pantograph	I	KCS
	872.3740	Retentive and splinting pin		EBL
	872.3750	Bracket adhesive resin and tooth conditioner	II	DYH, KZP
	872.3760	Denture relining, repairing, or rebasing resin		EBI
	872.3765	Pit and fissure sealant and conditioner		EBC
	872.3770	Temporary crown and bridge resin		EBG, POW
	872.3810	Root canal post	I	ELR
	872.3820	Root canal filling resin	II & III	KIF, NYD, MMT
	872.3830	Endodontic paper point	I	EKN
	872.3840	Endodontic silver point		EKL
	872.3850	Gutta percha		EKM
	872.3890	Endodontic stabilizing splint	II^a^	ELS
	872.3900	Posterior artificial tooth with a metal insert	I	ELJ
	872.3910	Backing and facing for an artificial tooth	I	ELK
	872.3920	Porcelain tooth	II	ELL
	872.3930	Bone grafting material	II^a^ & III.	LPK, LYC, NPK, NPL, NPM, NUN, NPZ, NQA
	872.3940	Total temporomandibular joint prosthesis	III	LZD
	872.3950	Glenoid fossa prosthesis		MPI
	872.3960	Mandibular condyle prosthesis		MPL
	872.3970	Interarticular disc prosthesis (interpositional implant)		MPJ
	872.3980	Endosseous dental implant accessories	I	NDP, NYE, OFY, QRQ
Subpart E - Surgical Devices	872.4120	Bone cutting instrument and accessories	II	DZH, DZI, DZJ, KMW, MXF, PLV, QRY
	872.4130	Intraoral dental drill	I	DZA
	872.4200	Dental handpiece and accessories		EBW, EFA, EFB, EGS, EKX, EKY, NYL
	872.4465	Gas-powered jet injector	II	EGQ
	872.4475	Spring-powered jet injector		EGM
	872.4535	Dental diamond instrument	I	DZP, NLD
	872.4565	Dental hand instrument		DZN, EAX, ECB, ECP, ECQ, ECR, ECS, ECT, EFK, EFL
	872.4600	Intraoral ligature and wire lock	II	DYX
	872.4620	Fiber optic dental light	I	EAY
	872.4630	Dental operating light		EAZ, EBA
	872.4730	Dental injecting needle		DZM, NMW
	872.4760	Bone plate	II	JEY, MDL, MQN
	872.4770	Temporary mandibular condyle reconstruction plate	II^a^	NEI
	872.4840	Rotary scaler	II	ELB
	872.4850	Ultrasonic scaler		ELC
	872.4880	Intraosseous fixation screw or wire		DZK, DZL
	872.4920	Dental electrosurgical unit and accessories		EKZ
Subpart F - Therapeutic Devices	872.5410	Orthodontic appliance and accessories	I	DYJ, DYO, DZC, DZD, ECI, ECM, ECN, ECO, EJF, NQS
	872.5470	Orthodontic plastic bracket	II	DYW, NJM, NLC, NXC, OYH, PLH, PNN
	872.5500	Extraoral orthodontic headgear		DZB
	872.5525	Preformed tooth positioner	I	DYT, KMY
	872.5550	Teething ring		KKO, MEF
	872.5560	Electrical salivary stimulatory system	II^a^	LTF, QTT
	872.5570	Intraoral devices for snoring and intraoral devices for snoring and obstructive sleep apnea		OZR, LQZ, LRK, OHP, ORY, PLC, MYB
	872.5571	Auto titration device for oral appliances		QCJ
	872.5580	Oral rinse to reduce the adhesion of dental plaque		NTO
Subpart G - Miscellaneous Devices	872.6010	Abrasive device and accessories	I	EEJ, EHJ, EHL, EHM, EJQ
	872.6030	Oral cavity abrasive polishing agent		EJR
	872.6050	Saliva absorber		EFN, KHR
	872.6070	Ultraviolet activator for polymerization	II	EBZ, QNF
	872.6080	Airbrush		KOJ, PIP
	872.6100	Anesthetic warmer	I	EFC, QGO
	872.6140	Articulation paper		EFH
	872.6200	Base plate shellac		EEA
	872.6250	Dental chair and accessories		KLC, NRU
	872.6290	Prophylaxis cup		EHK
	872.6300	Rubber dam and accessories		EEF, EIE, EJE, EJG
	872.6350	Ultraviolet detector	II	EAQ, NXV
	872.6390	Dental floss	I	JES
	872.6475	Heat source for bleaching teeth		EEG
	872.6510	Oral irrigation unit		EFS, OGT
	872.6570	Impression tube		KCQ
	872.6640	Dental operative unit and accessories		DYN, EBR, EHZ, EIA, NRD, OFX, QYJ
	872.6650	Massaging pick or tip for oral hygiene		JET, JEW
	872.6660	Porcelain powder for clinical use	II	EIH
	872.6670	Silicate protector	I	EFX
	872.6710	Boiling water sterilizer		ECG
	872.6730	Endodontic dry heat sterilizer	III	ECC, KOK
	872.6770	Cartridge syringe	II^a^	EJI
	872.6855	Manual toothbrush		EFW, LCN, MAU, MCF, NOB, NXZ, QJC, NSB
	872.6865	Powered toothbrush		JEQ, MMD, QIA
	872.6870	Disposable fluoride tray		KMT
	872.6880	Preformed impression tray		EHY
	872.6890	Intraoral dental wax		EGD, PFL

^a^ Special Control devices (FDA establishes special controls for Class II medical devices to provide reasonable assurance of the safety and effectiveness, where it is not sufficient under general control.).

^b^ Information extracted from CFR- Code of Federal Regulations Title 21 available at https://www.accessdata.fda.gov/scripts/cdrh/cfdocs/cfcfr/CFRSearch.cfm?CFRPart=872&showFR=1 accessed on Sept. 2025; Product Classification available at https://www.accessdata.fda.gov/scripts/cdrh/cfdocs/cfPCD/classification.cfm accessed on Sept. 2025.

Class I dental devices—such as dental mirrors (21 CFR 872.1430), explorers (21 CFR 872.1660), and examination gloves (21 CFR 880.6250)—are subject primarily to general controls, including establishment registration, device listing, labeling compliance, and adherence to Good Manufacturing Practices (GMPs). Most Class I devices are exempt from premarket notification [510(k)] requirements because their designs and materials are well established with predictable safety profiles. However, FDA inspections frequently identify noncompliance with the Quality System Regulation (QSR), including inadequate documentation, incomplete Device Master Records (DMRs), and lack of validated sterilization procedures. Even simple tools such as saliva ejectors or reusable explorers have been implicated in cross-contamination events when labeling or reprocessing instructions are unclear. Furthermore, the globalization of manufacturing and supply chains has introduced variability in production quality, especially among imported low-cost Class I instruments (100).

Class II dental devices, such as intraoral x-ray systems (21 CFR 872.1800), dental curing lights (21 CFR 872.6070), and air-driven handpieces (21 CFR 872.4200), present moderate risk and require both general and special controls to ensure safety and effectiveness. Most Class II devices are cleared through the 510(k) pathway by demonstrating substantial equivalence to a legally marketed predicate (21 CFR Part 807). Devices without a predicate may require a *de novo* classification request. Special controls for Class II devices can include biocompatibility testing under ISO 10993-1, electrical safety under IEC 60601, sterilization validation under ISO 11135 or ISO 17665, and software lifecycle documentation under IEC 62304 for digital systems. Design controls (21 CFR 820.30) are mandatory for Class II devices, including risk analysis, verification and validation, and traceability of design changes. Failures in software integration, sterilization, or labeling are common reasons for recalls.

Class III dental devices represent the highest risk category and include endosseous implants (21 CFR 872.3640), guided tissue regeneration membranes, bone grafting substitutes (21 CFR 872.3930), and combination products such as drug-eluting implants. These devices sustain or support human life or are critical to preventing major impairment of human health. As such, they require rigorous Premarket Approval (PMA) under 21 CFR Part 814, including clinical trials conducted under Investigational Device Exemption (IDE) protocols. Manufacturers must submit extensive bench, animal, and human data to demonstrate safety, performance, and biocompatibility (101). The FDA also mandates facility inspections to ensure QSR compliance under 21 CFR Part 820, including design validation, process control, complaint handling, and Corrective and Preventive Action (CAPA) systems. Class III devices with biologics or drug components, such as resorbable implants releasing bone morphogenetic proteins (BMPs), are regulated as combination products requiring coordination between the FDA's Center for Devices and Radiological Health (CDRH) and the Center for Biologics Evaluation and Research (CBER) (102).

U.S. dental device regulation aligns with international standards but demands rigorous compliance with statutory and quality requirements across all risk classifications. While Class I devices follow simplified regulatory pathways with limited premarket obligations, they still require strict oversight of manufacturing, labeling, and postmarket surveillance. In contrast, Class II and III devices necessitate extensive design controls, validation data, and clinical evidence to ensure safety and performance. As digital dentistry, additive manufacturing, and AI-driven technologies evolve, adherence to the FDA's Digital Health and Software as a Medical Device (SaMD) framework has become essential to maintain patient safety and global market access.

However, despite their therapeutic benefits, medical and dental devices can inadvertently contribute to antibiotic resistance. Devices that remain in contact with biological tissues—such as implants, catheters, and prosthetics—can develop biofilms that foster bacterial colonization and gene exchange, allowing pathogens to evade antimicrobial treatments. Repeated or prophylactic antibiotic use to control device-associated infections further drives multidrug resistance (103, 104). Regulatory frameworks mitigate these risks through stringent premarket evaluation, biocompatibility testing (ISO 10993), and sterilization validation (ISO 11135, ISO 17665), alongside mandatory compliance with the FDA's Quality System Regulation (21 CFR Part 820) and postmarket surveillance (21 CFR Part 803). By promoting antimicrobial stewardship, encouraging non-antibiotic surface technologies, and enforcing risk management principles under ISO 14971, regulators can reduce unnecessary antibiotic exposure and foster innovation in infection-resistant materials, supporting global efforts to curb antimicrobial resistance linked to medical and dental devices. Table 5 summarize dental device types, AMR/biofilm risks, and key regulatory controls.

**Table 5:** Summary of dental device types, AMR/biofilm risks, and key regulatory controls.

Device type (examples)	Typical AMR/biofilm risk	Key controls and evidence expectations
Implantable devices (implants, abutments, membranes)	Peri-implant biofilm persistence; difficult-to-treat infections; ARG exchange	Risk management (ISO 14971); biocompatibility (ISO 10993); clinically relevant antibiofilm endpoints; design controls; sterile barrier/sterilization validation when supplied sterile; post-market complaint and MDR monitoring
Reusable powered instruments (handpieces, ultrasonic scalers)	Internal contamination; aerosol-mediated transmission; reprocessing failures	Validated cleaning/sterilization and reprocessing IFU; process validation and labeling controls; routine maintenance and monitoring; design for cleanability
Dental unit waterlines (DUWLs)	Polymeric tubing biofilms; opportunistic pathogens; chronic low-level exposure	Waterline maintenance plans, periodic testing and disinfection; engineering controls; documentation within quality systems; clinic-level surveillance and corrective actions
Restorative materials and prosthetics (composites, crowns, CAD/CAM materials)	Biofilm accumulation at margins/interfaces; microleakage-associated infection risk	Material characterization and biocompatibility; performance testing under relevant biofilm models; labeling and clinical use conditions; change control for formulations/additives
Endodontic devices (files, obturation systems)	Biofilm carryover; contamination of complex geometries; retreatment-associated persistence	Sterilization validation for reusable components; single-use labeling where applicable; verification of cleaning for complex surfaces; surveillance of adverse events/complaints
Orthodontic appliances (brackets, aligners, retainers)	Plaque retention and dysbiosis; localized inflammation that can select for resistant taxa	Biocompatibility; usability and hygiene instructions; manufacturing quality controls; post-market feedback to refine materials and IFU

## 8 Challenges and opportunities

The increasing prevalence of MDR bacteria in the oral cavity poses a serious challenge to safe and effective dental practice, particularly during procedures involving implants, restorative materials, and endodontic devices. Pathogens such as methicillin-resistant *Staphylococcus aureus* (MRSA), vancomycin-resistant *Enterococcus faecalis*, extended-spectrum *β*-lactamase (ESBL)-producing *Klebsiella pneumoniae*, and *Pseudomonas aeruginosa* have been increasingly identified in peri-implant infections, deep periodontal lesions, and dental abscesses (105–108). Once these organisms colonize device surfaces, they form biofilms composed of microbial communities embedded in an extracellular polymeric matrix. Within biofilms, bacteria can exchange antibiotic resistance genes (ARGs) and reduce antibiotic penetration, resulting in resistance levels 100–1,000 times higher than in planktonic forms (109).

Excessive or prophylactic use of antibiotics in dental care further accelerates resistance by creating selective pressure that favors resistant strains. Inadequate infection control measures and limited AMR surveillance increase the risk of cross-contamination through contaminated dental unit waterlines (DUWLs), handpieces, and reusable instruments (110). These combined factors highlight the urgent need for improved infection control strategies and antimicrobial stewardship within dental practices.

Emerging advances in dental materials science and bioengineering offer new opportunities to reduce AMR associated with dental devices. Modern antimicrobial coatings, including silver-ion releasing surfaces, nitric oxide-emitting materials, and photocatalytic nanocomposites, are being explored for their ability to inhibit bacterial adhesion and early biofilm formation (111). Smart dental implants and restorative devices integrating biosensors capable of detecting biochemical markers—such as pH fluctuations, redox changes, or quorum-sensing molecules—may provide real-time monitoring and targeted therapeutic release (112). Furthermore, phage-based therapies and resorbable scaffolds impregnated with bacteriophages or antimicrobial peptides represent promising non-antibiotic alternatives for disrupting established biofilms without promoting bacterial resistance (103).

Regulatory agencies are increasingly supporting innovation in antimicrobial dental devices. The U.S. Food and Drug Administration (FDA) has expanded its Breakthrough Devices Program to facilitate the evaluation and approval of novel technologies addressing unmet clinical needs such as AMR-related infections (99). Similarly, the integration of rapid molecular diagnostic tools—including point-of-care qPCR, next-generation sequencing, and CRISPR-based assays—enables the identification of resistance genes before treatment, allowing more precise and personalized infection control approaches (99, 113).

Addressing MDR bacterial threats in dentistry requires a coordinated, multidisciplinary framework combining device innovation, microbiological surveillance, and regulatory compliance. Regulatory authorities such as the FDA and European Medicines Agency (EMA) increasingly accept submissions for novel antimicrobial materials when supported by strong preclinical, clinical, and postmarket data. Manufacturers must comply with standards such as ISO 13485, ISO 10993, and ISO 14971, while ensuring validated antimicrobial performance through standardized testing. By aligning device innovation with antimicrobial stewardship and international regulatory frameworks, the dental industry can play a vital role in reducing infection risks, improving patient safety, and mitigating the global spread of antibiotic resistance.

## 9 Conclusion

The rise of AMR presents a critical challenge to modern dentistry, as biofilm-forming microorganisms increasingly compromise the safety and efficacy of dental devices and treatments. The oral microbiome, while essential to maintaining homeostasis, can act as a reservoir for ARGs, enabling the persistence and spread of MDR pathogens on implant and restorative surfaces. Innovative strategies such as AMP coatings, silver nanoparticle-based materials, and smart, stimuli-responsive surfaces have demonstrated potential to prevent bacterial adhesion and biofilm formation while supporting tissue integration. Nonetheless, the success of these technologies depends on rigorous regulatory compliance and quality assurance guided by frameworks like FDA 21 CFR 820, ISO 13485, and ISO 14971. A coordinated approach that integrates materials science, microbiology, clinical practice, and regulatory oversight is vital to advance infection-resistant, biocompatible dental devices. Strengthening antimicrobial stewardship, standardizing sterilization procedures, and promoting global regulatory harmonization will be essential to curb AMR emergence and ensure long-term patient safety in dental care.
